# Pathophysiology, Management, and Outcome of Persistent Pulmonary Hypertension of the Newborn: A Clinical Review

**DOI:** 10.3389/fped.2013.00023

**Published:** 2013-09-02

**Authors:** Mohammed Puthiyachirakkal, Maroun J. Mhanna

**Affiliations:** ^1^Department of Pediatrics, Division of Neonatology, Case Western Reserve University at MetroHealth Medical Center, Cleveland, OH, USA

**Keywords:** persistent pulmonary hypertension, newborn, pathophysiology, outcome, treatment

## Abstract

Persistent Pulmonary Hypertension of the Newborn (PPHN) results from the failure of relaxation of the pulmonary vasculature at birth, leading to shunting of non-oxygenated blood from the pulmonary to the systemic circulation. More often, full term and near-term infants are affected, however it is not uncommon to see PPHN in preterm infants who have respiratory distress syndrome. In some infants pulmonary vascular remodeling is present at birth, pointing toward the prenatal onset of the disease process. Regardless of the etiology, PPHN should be diagnosed and treated as soon as possible to avoid hypoxia related short term and long-term morbidities. The mainstay therapy is the treatment of the underlying condition along with several promising therapeutic modalities such as oxygen supplementation, mechanical ventilation, nitric oxide, phosphodiesterase inhibitors, prostaglandins analogs, endothelin receptor antagonists, and extracorporeal membrane oxygenation. The optimal approach to the management of PPHN remains controversial. After discharge from the NICU, infants with PPHN warrant long-term follow up since they are at risk for neurodevelopmental disabilities and chronic health conditions.

## Introduction

Intra uterine relative hypoxemia, along with a multitude of other factors is responsible for an elevated pulmonary vascular resistance (PVR) that is almost twice as high in a fetus as in a newborn infant. Soon after birth, there is an improvement in ventilation, oxygenation, and pulmonary blood flow that lead to a decrease in PVR in newborn infants. The neonatal PVR gradually decreases after birth to reach the adult levels by 2 weeks of age (1). A failure of the post natal reduction in PVR results in a serious medical condition called Persistent Pulmonary Hypertension of the Newborn (PPHN). Unless identified and treated promptly, PPHN is associated with a significant morbidity and mortality that can be as high as 10–20% (1). The pathogenesis of PPHN is multi-factorial including maternal and neonatal causes; However Meconium Aspiration Syndrome (MAS) remains the most common cause responsible for PPHN (2). The introduction of Nitric Oxide (NO) as a therapeutic modality has revolutionized the management of PPHN and successfully reduced the need for Extracorporeal Membrane Oxygenation (ECMO); however it did not reduce overall mortality in these infants (3). This article reviews our current understanding of the pathophysiology of PPHN, the current available therapeutic modalities, and the short and long-term outcome of newborn infants with PPHN.

## Methods

In January of 2013, EMBASE and PubMed databases were searched using the key words persistent pulmonary hypertension, newborn, inhaled NO, and ECMO. Cross-references of the relevant articles were also searched for additional studies. No restrictions on language or study designs were applied. Adult and pediatric clinical studies as well as animal and bench research studies were reviewed in preparation for this review. The quality of evidence of several key clinical studies, included in this review, was graded according to the American College of Chest Physicians (ACCPs) grading guidelines which is a modified grading system of the international GRADE group. The grading system classifies recommendations as strong (Grade 1) or weak (Grade 2), and quality of evidence as high (Grade A), moderate (Grade B), or low (Grade C) according to the study design, the consistency of the results, and the directness of the evidence (4).

## Fetal Circulation and Neonatal Transition

**Figure 1:**
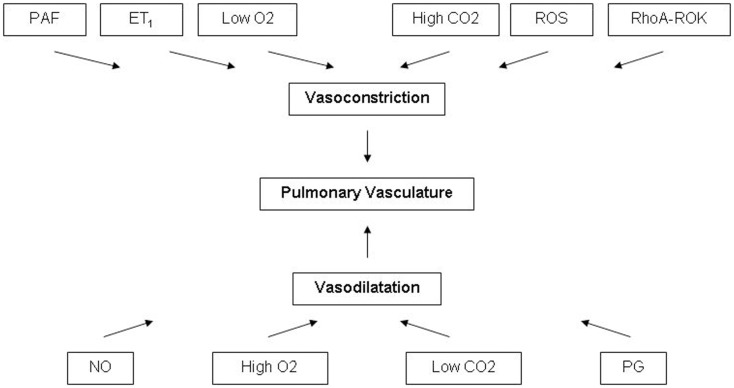
**Factors affecting the pulmonary vasculature tone**. PAF, platelet activating factor; ET1, endothelin-1; ROS, reactive oxygen species; RhoA-ROK, Rho A-Rho kinase; O_2_, oxygen; CO_2_, carbon dioxide; NO, nitric oxide; PG, prostaglandins.

The oxygenation of the human fetus is placenta dependent. Oxygenated blood is carried by the umbilical vein to the right atrium and is preferentially carried to left atrium through the foramen ovale. The portion of inferior vena cava distal to the diaphragm receives the blood from ductus venosus, portal vein, right, and left hepatic veins. A membranous valve called Eustachian valve preferentially direct blood from most saturated ductus venosus to the left atrium (5). The venous return from the superior vena cava and inferior vena cava are mixed with the umbilical venous blood in the right atrium and then enters to the right ventricle. The majority of the right ventricular output is directed through the ductus arteriosus to the descending thoracic aorta, and only 13–25% of the right ventricular output is directed to the pulmonary vasculature (1). Therefore the saturation in the ascending aorta, descending aorta, pulmonary artery, and vein are 65, 55, 55, and 45%, respectively (1). As gestation advances, the weight indexed PVR increases. Factors maintaining a high pulmonary vascular tone are multiple including fetal lung fluid, low oxygen tension, and vasoactive factors such as endothelin-1 (ET_1_), platelet activating factor (PAF), reactive oxygen species (ROS), and increased Rho A-Rho Kinase (RhoA-ROK) signaling (1). ET_1_ is the predominant ET in the pulmonary vascular endothelial cells. Under hypoxic condition ET_1_ binds to its receptors ET_A_ and ET_B_ receptors. Stimulation of ET_A_ causes vasoconstriction by elevating intracellular Ca and stimulation of ET_B_ causes vasodilatation (1). The hypoxic fetal condition also inhibits the production of vasodilator factors such as NO and prostaglandins (PGs); Figure 1 (1, 6).

At birth with the first breath the lungs are filled with air and there is an abrupt increase in pulmonary blood flow, creating a shear stress with in the vessel wall. Shear stress and oxygenation stimulate endothelial nitric oxide synthase (eNOS) and up regulate its expression. As a result NO is synthesized from L-Arginine which then diffuses to the pulmonary smooth muscle cells and activates soluble Guanylyl cyclase (sGC). Oxygenation up regulates the expression and activity of sGC which converts GTP to cGMP which further through the activity of cGMP dependent protein kinase (PKG) cause smooth muscle relaxation. Oxygenation also inhibits the enzymatic activity of phosphodiesterase 5 (PDE5) which converts cGMP to 5′ cGMP (1, 6).

Another pathway of pulmonary vasodilatation is through the production of PG by the endothelium. The oxygenation is the stimulus for the production of PG from the membrane arachidonic acid (AA) with cyclooxygenase (COX) as the rate limiting enzyme. The predominant PG is PGI2 which stimulates adenylate cyclase resulting in conversion of ATP to cAMP, causing relaxation of the pulmonary vasculature through cAMP dependent protein kinase (PKA). Phosphodiesterase 3 (PDE3) enzyme converts cAMP to AMP (1, 6). Overall the decrease in PVR caused by PG is less than with NO (1).

In addition to NO and PGI2, potassium channel and calcium channels are also involved in pulmonary vasodilatation. Oxygenation results in pulmonary dilatation through the activation of K channels and reduction in Ca channels in pulmonary artery smooth muscles (1).

## Pathogenesis

The PPHN is more common in late preterm and term babies. The incidence of PPHN in term and late preterm is estimated to be 1–2/1000 (2, 7). There are three main types of PPHN related to under development, mal-development, and mal-adaptation (7, 8). Some of the causes of PPHN related to underdevelopment include congenital diaphragmatic hernia (CDH), pulmonary hypoplasia, oligohydramnios from premature rupture of membranes or renal agenesis, pleural effusion, vascular anomalies, asphyxiating thoracic dystrophy, phrenic nerve agenesis, and alveolar capillary dysplasia (7). Some of the causes related to mal-development include idiopathic or Primary PPHN, chronic fetal hypoxia, fetal anemia, and premature closure of the ductus arteriosus (7). Some of the causes of PPHN related to mal-adaptation include asphyxia, MAS, neonatal respiratory distress syndrome, and sepsis/pneumonia (7). Congenital heart diseases such as total anomalous pulmonary venous return, left atrial, or mitral obstruction, and hypoplastic left heart syndrome can be responsible for PPHN. Polycythemia, hypoglycemia, and hypocalcemia can also be responsible for PPHN. However MAS is the most common cause of PPHN (2). Pulmonary hypertension in MAS results from airway obstruction, inactivation of surfactant, and chemical pneumonitis from the release of proinflammatory cytokines (9). Pneumothorax, change of fetal heart beat pattern and asphyxia are also risk factors for PPHN in MAS (10). The pulmonary arteries in neonates with idiopathic PPHN are characterized by the proliferation of smooth muscle cells of the normally muscle free peripheral arteries. Animal studies have shown that hypoxia result in decreased activity of matrix metalloproteinase-2 (MMP-2) which is responsible for the deposition of collagen and elastin in the arterial adventitia. As a result of smooth muscle proliferation and adventitial thickening the PVR increases to suprasystemic levels causing right-to-left shunting through the ductus arteriosus and/or the foramen ovale. The resulting hypoxemia and acidosis further aggravate the pulmonary vasoconstriction, creating a vicious cycle of right-to-left shunting, hypoxia, and acidosis (1, 6, 11).

## Risk Factors

Epidemiological studies have shown that the prevalence of PPHN is increased in male infants, black or Asian maternal race, pre-conception elevated body mass index (BMI > 27 vs. BMI < 20), diabetes, asthma, cesarean section, late preterm, and large for gestational age (LGA); Table 1 (12, 13). Other risk factors have also been described including histological chorioamnionitis and or funisitis (14), and use of selective serotonin reuptake inhibitors (SSRIs) (15, 16). In a fetal pulmonary artery Doppler velocity study, SSRI exposure was found to increase slightly the right pulmonary arterial blood flow during the third trimester; however not to the extent of mounting PPHN (Grade 2C) (17). Another risk factor is the antenatal exposure to non-steroidal anti-inflammatory drugs (NSAIDs; Grade 2C) (18). Animal studies have shown that exposure to PG inhibitors will lead to histological features similar to PPHN (Grade 2C) (18). In one study, the use of NSAIDs during pregnancy, particularly aspirin, ibuprofen, and naproxen were found to be significantly associated with PPHN (19). However in a large epidemiological study conducted in four metropolitan areas (Boston, Philadelphia, San Diego, and Toronto) between 1998 and 2003 NSAIDs were not found to be associated with PPHN (18). Group B *Streptococcus* (GBS) infection continues to be the predominant cause of early onset sepsis. The Phosphatidylglycerol and cardiolipin are the dominant phospholipids of GBS that are associated with PPHN; and animal studies have shown that indomethacin can lower the PVR induced by these agents (20). There are case reports of ureaplasma infection causing PPHN with a high case fatality rate, and Phospholipase A and C as the incriminating agents (21). Following the introduction of therapeutic hypothermia for hypoxic ischemic encephalopathy there was a concern whether cooling will aggravate PPHN. But none of the studies have shown association of cooling with PPHN even though some pulmonary dysfunction can happen in babies while on cooling therapy (22, 23).

**Table 1:** **Potential risk factors for the development of PPHN**.

Male gender
African or Asian maternal race
Pre-conception maternal overweight
Maternal diabetes
Maternal asthma
Cesarean section
Late preterm and large for gestational age
Chorioamnionitis
Antenatal exposure to selective serotonin reuptake inhibitors
Antenatal exposure to non-steroidal anti-inflammatory drugs
Infection (mainly Group B *Streptococcus*)
Hypothermia
Hypocalcemia
Polycythemia

## Diagnosis

Persistent pulmonary hypertension of the newborn is suspected clinically in term and near-term infants who have variable hemoglobin oxygen saturation. A hyperoxia test, a quick bedside test, can be performed to help making the diagnosis of PPHN. The test consists of exposing infants to a Fraction of inspired Oxygen (FiO_2_) of 100% for 5–10 min, and obtaining an arterial blood gas (ABG). If the ABG shows that the partial arterial pressure of Oxygen (PaO_2_) is<150 torr, the diagnosis of PPHN or a cyanotic heart disease are suspected. To rule out a structural or congenital heart disease, an echocardiogram is indicated. Among the various methods used to measure the pulmonary pressure, the pressure drop across the PDA is a reliable method followed by the measurement of TR (tricuspid regurgitation) jet velocity. However TR jet velocity has limitation in the presence of right ventricular dysfunction which may be a feature of severe PPHN. In the absence of TR jet, systolic ventricular septal flattening may be useful to diagnose PPHN which indicate whether the right ventricular pressures are>half or<half of left ventricular systolic pressure. Echocardiographic findings of impaired ejection fraction and stroke volume carry a poor prognosis (24, 25). Imaging studies can be valuable to make the diagnosis of PPHN, a chest X-ray can show oligemic lung fields in primary PPHN and be helpful in diagnosing lung disease. A blood work up is helpful to identify the etiological factors responsible for PPHN, a complete blood count can show abnormal white blood cell counts and help rule out infections and can show polycythemia. Blood glucose and calcium level are helpful to rule out metabolic causes. An ECG is not usually helpful for the diagnosis. Brain natriuretic peptide (BNP) is a reliable and easily available marker which contributes to the diagnosis of PPHN especially if an echocardiogram is not readily available. Reynolds et al. in a prospective cohort study have shown that an initial BNP level of 550 pg/ml or greater was predictive of PPHN with a sensitivity of 85% and a specificity of 100% (Grade 2C) (26). In a prospective cohort study, Vijlbrief et al. have shown that BNP level significantly increases in patients with rebound pulmonary hypertension following the discontinuation of NO (27).

## Management

The management of PPHN is to restore the cardiopulmonary adaptation without inflicting an iatrogenic pulmonary injury (Table 2). The management entails the treatment of the underlying etiology, maintenance of a normal systemic blood pressure, and provision of an adequate tissue oxygenation.

**Table 2:** **Treatment of PPHN**.

Treatment of the underlying etiology
Mechanical ventilation
Surfactant
Nitric oxide (NO)
Phospho diesterase enzyme (PDE) inhibitors
• PDE_5_ inhibitor: sildenafil
• PDE_3_ inhibitor: milrinone
Prostaglandin analogs (PG)
• PGI2: iloprost
Magnesium sulfate
Endothelin receptor (ETR) antagonists
• ETR: bosentan
Extra corporeal membrane oxygenation (ECMO)

The infant’s body temperature should be stabilized and handling should be minimized to avoid stimulation and agitation of the newborn infant. The use of sedatives is indicated especially in infants who are mechanically ventilated to avoid agitation and asynchrony with the ventilator support. The blood pressure should be maintained with fluid resuscitation, pressors, and inotropes. Antimicrobial therapy is indicated if infection is suspected.

Oxygen is a very important pulmonary vasodilator. If adequate oxygenation cannot be attained by nasal cannula or hood oxygen, infant should be ventilated. Hyperventilation used to be a common practice to induce a respiratory alkalosis and therefore a decrease in the PVR (28). But care should be taken to avoid ventilator induced lung injury as it can worsen the pulmonary arterial pressure (28). In addition, hyperventilation induced alkalosis that can be responsible for shifting the oxygen hemoglobin dissociation curve to the left compromising oxygen delivery to the tissues. There has been strong association between partial arterial carbon dioxide (PaCO_2_) levels<25–30 mmHg and an increased incidence of cystic periventricular leukomalacia (PVL) and cerebral palsy (CP) in preterm and near-term infants (7). Therefore mechanical ventilation should be targeting PaCO_2_ levels of 40–60 mmHg and a PaO_2_ of 60–90 mmHg. High frequency oscillatory ventilation (HFOV) can be attempted to minimize lung injury. However a recent meta analysis have failed to show a clear benefit of HFOV over conventional ventilation as an elective or as a rescue mode of ventilation in term or preterm infants with PPHN (2, 7, 25). During ventilation and respiratory support, a special attention should be taken to avoid hyperoxia; since hyperoxia has been shown to cause a significant lung injury as it has been shown in multiple animal models and it can increase the development of peroxynitrite in the presence of NO (1).

## Surfactant

Surfactant therapy in neonates with PPHN has had variable results. Surfactant did not improve lung compliance or duration to extubation in infants with CDH treated with ECMO; however it improved the severity of pulmonary morbidity, air leaks, and length of hospital stay in infants with MAS and pneumonia (7). In a multicenter randomized controlled study comparing surfactant to placebo, surfactant use significantly decreased the need for ECMO (Grade 1C) (7, 29). In another study, surfactant lung lavage (SLL) showed an improvement in oxygenation, a decrease in mean airway pressure (MAP), and A-a gradients, however it did not improve the duration of mechanical ventilation, the use of inhaled NO, length of hospitalization, or overall complications (Grade 2A) (30).

## Nitric Oxide

Several randomized controlled trials have demonstrated the effectiveness of NO as a selective pulmonary vasodilator without many side effects if used at proper concentration (Grade 1B) (3). NO can be started in term or late preterm infants when the oxygen index (OI) exceeds 25 or when the PaO_2_, while receiving 100% FiO_2_, is<100 mmHg. In one of the early randomized controlled study, the Neonatal Inhaled Nitric Oxide Study (NINOS), the most effective dose of NO was 20 ppm. However few patients (6%) who did not respond to 20 ppm responded to 80 ppm. A higher concentration of NO is not without side effects. A high dose of NO is associated with methemoglobinemia. In a recent trial, a concentration of 80 ppm of NO was associated with methemoglobinemia at a concentration of 7% and higher in 35% of the time (31). In patients who do not respond to 20 ppm of NO, a brief exposure to higher doses (40, 80 ppm) can be attempted, however a rigorous monitoring of the concentration of methemoglobinemia should be undertaken. NO has been shown to reduce the need for ECMO by 40% but it has not been able to reduce mortality (Grade 1B) (3). Meta analysis of randomized controlled trials of inhaled NO in term and near-term infants did not show the beneficial effects of NO in patients with pulmonary hypertension secondary to CDH (Grade 1B) (3). NO seems to be equally effective in moderate or severe illnesses. In premature babies with hypoxemic respiratory failure NO therapy was shown to improve oxygenation without improvement in survival; and it was associated with an increased risk for IVH (Grade 1B) (3). Infants treated with NO may not respond to the gas for multiple reasons, including the inability to deliver NO to the pulmonary circulation secondary to poor lung inflation, myocardial dysfunction, systemic hypotension, severe pulmonary vascular structural disease, missed anatomic cardiovascular lesions, such as total anomalous pulmonary venous return, coarctation of the aorta, and alveolar capillary dysplasia (Grade 1B) (3, 32). A prospective cross-sectional online survey of neonatologists regarding the management of PPHN has shown that wide variation exists on the threshold for initiation of iNO, dosage, and weaning strategy. The most common initiating dose of iNO by neonatologists is 20 ppm and 25% of neonatologist use more than the maximum recommended dose of 20 ppm (33). The response to NO is defined as an improvement in PaO_2_ or reduction in FiO_2_ (33).

## Other Medications

Several medications have been used in the treatment of PPHN including Phospho Diesterase Enzyme (PDE) inhibitors, PDE_5_ inhibitors, PDE_3_ inhibitors, Endothelin receptor (ETR) antagonists, PGs, Calcium channel blockers, magnesium sulfate, scavengers of ROS, and RhoA/Rho kinase inhibitors.

## PDE_5_ Inhibitors

Sildenafil is the only PDE_5_ inhibitor that is available for use in newborn infants with PPHN. Sildenafil causes pulmonary vasodilatation by increasing the availability of cGMP. In a recent meta-analysis sildenafil was shown to reduce mortality in newborn infants where NO was not available (RR 0.20, 95% CI 0.07–0.57) without clinically important side effects (Grade 2A) (34). In an open-label, dose-escalation trial in newborns with PPHN continuous IV infusion of sildenafil, acute, and sustained improvements in oxygenation was noted (35). Sildenafil is associated with side effects in infants. It can cause systemic hypotension and retinopathy of prematurity in preterm infants. Kehat et al. in a case series of 22 full terms and near-term infants did not find any ocular complication related to sildenafil use (36).

## PDE_3_ Inhibitors

Milrinone is a PDE_3_ inhibitor used for the treatment of PPHN in infants. Milrinone increases the availability of cAMP causing positive inotropic effect, peripheral vasodilatation, left ventricular afterload reduction, and therefore increasing the cardiac output. There are no randomized controlled studies assessing the efficacy and safety of milrinone in infants with PPHN (37). More studies are needed before routinely recommending milrinone in infants with PPHN.

## Prostaglandin Analogs

Intravenous PGs can cause both systemic and pulmonary vasodilatation. Randomized trials in adults and animal models have shown its efficacy; however in neonates only case reports are available. Case reports have shown that inhaled/nebulized PGI_2_ and its analogs iloprost can be helpful in newborn infants (38).

## Magnesium Sulfate

Animal studies have shown that intravenous magnesium sulfate can cause reduction in pulmonary artery pressures (38). However in newborn infants, only observational studies are available showing that magnesium sulfate can be helpful (38). The use of magnesium sulfate should be limited especially that it can be associated with systemic hypotension.

## Endothelin Receptor Antagonists

Bosentan is a non-selective ETR antagonist; Sitaxentan and ambrisentan are selective ET_A_ receptor inhibitors. Randomized controlled studies and systematic reviews in adults have shown improvement in outcomes of patients with pulmonary hypertension (38). Nakwan et al. have reported the benefits of bosentan in neonates with PPHN (39). A recent randomized controlled trial has shown its efficacy over placebo in a setting where NO was not available (Grade 2B) (40).

## Extracorporeal Membrane Oxygenation

Extracorporeal membrane oxygenation is the ultimate rescue therapy for infants with PPHN with an oxygenation index persistently>40 despite treatment with NO and optimal ventilator management. To be candidates for ECMO, infant should be>2 kg in weight, and should have no contraindication to heparinization such as severe IVH or non-survivable congenital anomalies. Overall the survival of neonates with PPHN after ECMO therapy is 80% and venoarterial ECMO is most often used for ease of access (41). There are few predictors of good outcome in infants with PPHN requiring ECMO. Higher birth weight, higher 5-min Apgar score, absence of CDH, and postnatal diagnosis of CDH are associated with an improvement in survival (41). The most common complication of ECMO therapy is cardiovascular followed by mechanical and renal. Neurodevelopmental disability can be as common as 15–20% among ECMO survivors. The rate of complications increases as the days on ECMO increases (41).

## Outcome

The long-term outcome of infants with PPHN may depend on their underlying conditions and the therapeutic interventions that they have received at birth. The rate of neurodevelopmental disabilities including cognitive delays and hearing deficit can be seen in 6.4% of PPHN survivors (42). Feeding problems and short term respiratory morbidities can be seen also in 24% of PPHN survivors (42). Rosenberg et al. found no differences in medical, neurodevelopmental, and social/emotional/behavioral outcomes at school age, between children with PPHN who were treated with iNO, with or without ECMO, and infants who were treated without exposure to iNO (42). In a study at 18–24 months of age, inhaled NO was not associated with an increase in neurodevelopmental, behavioral, or medical abnormalities in infants who were treated for PPHN with or without iNO (43). In another long-term follow up study, Eriksen et al. found that infants, who were treated for PPHN at birth, had a higher prevalence of sensorineural hearing loss, chronic health problems, need for bronchodilator therapy, and remedial education at the age of 5–10 years in comparison to their controls (Grade 2C) (44). Long-term medical and neurodevelopmental follow up of infants with PPHN is warranted.

## Conclusion

Persistent pulmonary hypertension of the newborn is a neonatal emergency that requires early intervention and treatment to prevent severe hypoxia and various short term and long-term morbidities. The mainstay therapy is the treatment of the underlying condition along with several promising therapeutic modalities such as oxygen supplementation, mechanical ventilation, NO, PDE inhibitors, PG analogs, ET receptor antagonists, and ECMO. However the optimal approach to the management of PPHN remains controversial. Future high quality randomized controlled studies of existent and new therapeutic modalities are needed to develop strong, evidence based guidelines for the management of PPHN. After discharge from the NICU, infants with PPHN warrant long-term follow up since they are at risk for neurodevelopmental disabilities and chronic health conditions.

## Conflict of Interest Statement

The authors declare that the research was conducted in the absence of any commercial or financial relationships that could be construed as a potential conflict of interest.
